# Effects of probiotic treatment on patients and animals with chronic obstructive pulmonary disease: a systematic review and meta-analysis of randomized control trials

**DOI:** 10.3389/fcimb.2024.1411222

**Published:** 2024-09-11

**Authors:** Ziying Su, Chenxi Ma, Xiaosong Ru, Sijia Zhang, Chuyi Wu, Yue Huang, Huijie Cen, Zihui Yin, Jianping Zhang

**Affiliations:** ^1^ The First School of Clinical Medicine, Zhejiang Chinese Medical University, Hangzhou, China; ^2^ The Second School of Clinical Medicine, Zhejiang Chinese Medical University, Hangzhou, China; ^3^ The Third School of Clinical Medicine, Zhejiang Chinese Medical University, Hangzhou, China; ^4^ School of Basic Medical Sciences, Zhejiang Chinese Medical University, Hangzhou, China

**Keywords:** probiotics, chronic obstructive pulmonary disease, inflammatory changes, forced expiratory volume in one second, deposition of pulmonary collagen fibers, meta-analysis

## Abstract

**Objective:**

In recent years, the lung-gut axis has received increasing attention. The oxidative stress and systemic hypoxia occurring in chronic obstructive pulmonary disease (COPD) are related to gut dysfunction. That suggests probiotics have a potential therapeutic role in COPD. In this study, we therefore evaluated the ameliorative effects of probiotics on COPD.

**Methods:**

Searches were conducted in four electronic databases, including PubMed, Cochrane Library, the NIH clinical registry Clinical Trials. Gov and EMBASE. The data extracted was analyzed statistically in this study using StataMP17 software, with mean difference (MD) chosen as the effect size for continuous variables, and the results expressed as effect sizes and their 95% confidence intervals (CIs). Standardized Mean Difference (SMD) was used if the data units were different.

**Results:**

We included three randomized, controlled, double-blind clinical trials and five randomized controlled animal studies. The results show that for lung function, probiotics improved %FEV1 in COPD patients (MD = 3.02, 95%CI: 1.10, 4.93). Additionally, in inflammation, probiotics increased IL-10 (SMD = 1.99, 95%CI: 1.02, 2.96) and decreased inflammatory markers such as TNF-α (SMD= -2.64, 95%Cl: -3.38, -1.90), IL-1β (SMD= -3.49, 95%Cl: -4.58, -2.40), and IL-6 (SMD= -6.54, 95%Cl: -8.36, -4.73) in COPD animals, while having no significant effect on C-reactive protein (MD = 0.30, 95%CI: -0.71, 1.32) in COPD patients. For lung structure, probiotics significantly reduced the degree of pulmonary collagen fibers deposition in COPD animals (SMD = -2.25, 95%CI: -3.08, -1.41).

**Conclusion:**

Overall, probiotics may be an additional approach that can improve COPD. Further clinical trials are needed to evaluate the efficacy, safety, and impact factors of probiotics for COPD.

**Systematic Review Registration:**

https://inplasy.com/inplasy-2023-4-0023/, identifier INPLASY202340023.

## 1 Introduction

The characteristics of chronic obstructive pulmonary disease (COPD) include persistent respiratory symptoms and airflow limitation, mostly caused by high exposure to toxic particles or gases and occurring under the combined influence of genetic, developmental and social factors (Christenson et al., 2022). As a growing public health problem, COPD accounted for approximately 55% of the prevalence of chronic respiratory diseases in men and women in 2017 and represents a relative increase of 5.9% compared to the overall prevalence in 1990 (Collaborators, G. C. R. D, 2020).

In 2019, the World Health Organization reported it as the third leading cause of death worldwide (Collaborators, G. C. R. D, 2020). As the population ages (Mathers and Loncar, 2006), the prevalence of COPD will continue to grow, which not only reduces the quality of life, but also creates a huge health, social, and economic burden (Stolz et al., 2022). However, the effectiveness of relevant treatments is limited, in which drug side effects, especially inhaled glucocorticoids, may exacerbate the risk of pneumonia (Celli and Wedzicha, 2019). Therefore, there is an urgent need to find an alternative treatment to improve COPD.

The gut microbiome has been identified as a crucial element influencing lung health, including lung physiology and function, and immunity (Bulanda and Wypych, 2022). “Lung-gut axis” illustrates how gut microbes and respiratory system interact. This interaction in COPD may crosstalk bidirectionally through the major pathway of systemic inflammation (Wang et al., 2023). It has been shown that changes in gut microbiota composition, such as fecal microbiota transplantation and probiotic supplements, may have a positive or negative effect on lung function in recent years (N. Li et al., 2021; Jamalkandi et al., 2021; Lai et al., 2022). Probiotics are defined as live microorganisms that, when ingested in sufficient amounts, produce health benefits for the host (Suez et al., 2019; Freedman et al., 2020). Relevant studies have shown that probiotics can influence the development of COPD by altering the gut microbiota (Yu et al., 2023).

However, there is a paucity of relevant data and inconsistencies in the results of trials, although some trials have reported that probiotic supplementation improves COPD or prevents COPD exacerbations (Jamalkandi et al., 2021; Olímpio et al., 2023). The specific efficacy of probiotics for treating COPD in animals and humans has not been clearly clarified (Bikov et al., 2022). To date, relevant meta-analyses have not been published. Therefore, we included as many studies as possible, including animal experiments, because animals can be molded to approach the disease state of COPD in humans after tobacco smoke inhalation, lipopolysaccharide (LPS) injection, and diesel exhaust particulate (DEP) exposure. The aim of our meta-analysis was to synthesize relevant literature data to quantitatively assess the effectiveness of probiotic treatment of COPD in humans and animals.

## 2 Methods

The study is based on a program prospectively registered on the INLASY platform (registration number: INPLASY202340023) (Su et al., 2023) and this report follows the PRISMA statement (Page et al., 2021).

### 2.1 Methods for conducting literature searches

The literature was searched systematically to discover studies regarding probiotics’ effectiveness in treating COPD. Searches were conducted in four electronic databases, including PubMed, Cochrane Library, the NIH clinical registry Clinical Trials. Gov and EMBASE. We used medical subject headings and free text terms such as “probiotic” or “bifidobacterial” or “Lactobacillales” or “microorganism” or “synbiotic” and “chronic obstructive pulmonary disease” to find relevant articles published before August 2024. The language of study was limited to English. And the articles retrieved included both animal and human studies.

### 2.2 Criteria for inclusion and exclusion

Each of the included studies strictly met the requirements of the proposed PICOS framework. PICOS contains Population, Intervention, Comparison, Outcome, and Study Design. Z.S. and X.R. screened the literature independently using EndNote software. Any disputes were resolved through third-party negotiation.

The standards for human research are as follows:

1. Randomized controlled trial.
2. Patients meeting any recognized diagnostic criteria for COPD will be included.
3. Studies including pregnant women, patients with gastrointestinal disorders or those who have undergone gastrointestinal surgery are excluded.
4. There were no limitations based on age, gender, or race/ethnicity.
5. The intervention in the experimental group consisted of oral probiotics, regardless of dose, frequency.
6. The control group had the same conditions as the experimental group, except that there was no probiotic intervention.
7. There were measurements of improvement in COPD, such as changes in lung function and inflammatory markers.

The standards for animal research are as follows:

1. Randomized controlled trial.
2. Study subjects were rodents that were modelled to resemble a state of COPD.
3. The intervention in the experimental group consisted of oral probiotics, regardless of dose, frequency.
4. Controls were in the same conditions as the experimental group, except for the absence of probiotic interventions.
5. Outcomes of the study consisted of structural and functional changes in the lungs related to COPD, such as indicators of inflammation.

### 2.3 Data extraction

The information pertaining to the studies included was extracted, including author names, country of origin, year of publication, details of the target population, probiotic implementation specifics, and outcomes. Z.S. and C.M. performed this task independently. In animal studies, information on the species used and the modelling methods employed was included. In human studies, we also added COPD duration and grade.

For each study, the mean, standard deviation, and sample size were extracted for each group. When we could not obtain these data, we asked the authors to provide unpublished data. When data were presented in a non-specific form in graphs and tables, we used numerical scales to estimate the data from them (Song et al., 2017; Xie et al., 2014). After the data were rigorously estimated, statistical methods were used to calculate their mean and standard deviation (Shi et al., 2023, 2020; Luo et al., 2018; Wan et al., 2014).

### 2.4 Risk-of-bias and assessing quality

Z.S. and C.M. evaluated the potential for bias using the Cochrane collaboration tool (Higgins et al., 2011). The risks were rated “low”, “high” or “unknown”. Any disputes were resolved through third-party negotiation. By an assessment of performance, selection, detection, reporting and attrition biases, we clarified the quality of included studies.

### 2.5 Analysis and synthesis of data

The data extracted was analyzed statistically in this study using StataMP17 (64-bit) software, with Mean Difference (MD) chosen as the effect size for continuous variables, and the results expressed as effect sizes and their 95% confidence intervals (CIs). SMD (Standardized Mean Difference) was used if the data units are different. In terms of interpreting the results, if the final combined result of the forest plot was situated on the left side of the x-axis, this indicated a reduction in the corresponding indicator; conversely, if it was located on the right side of the x-axis, this signified an increase in the corresponding indicator.

Before combining the effect sizes, the included studies were tested for heterogeneity so as to determine whether there was any homogeneity among the studies. The Cochran’s Q test and I^2^ statistic, combined with the overlap of the confidence intervals, were used to measure the magnitude of heterogeneity. Studies were judged to be homogeneous when P > 0.1, I² < 50%, and the overlap of the confidence intervals was large. Conversely, p < 0.1, I² > 50%, and a small overlap of confidence intervals indicate heterogeneity across studies. When the studies were homogeneous, we used fixed effect model for data analysis. If not, we used a random effect model. Moreover, descriptive analyses were used when the source of the data was not known or when there was too much heterogeneity among the studies.

## 3 Results

### 3.1 Features of the study

**Figure 1:**
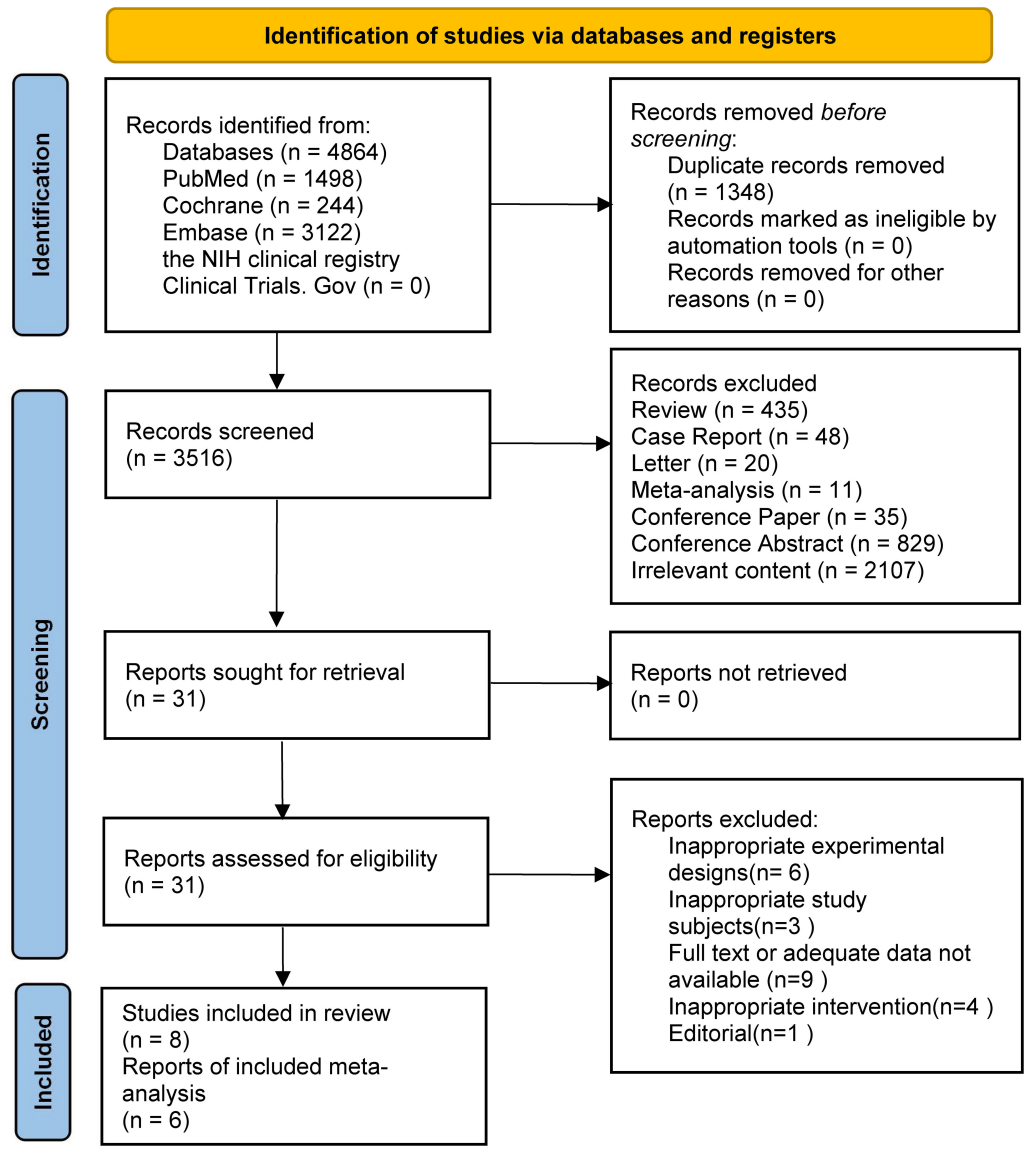
PRISMA flow diagram.

The search strategy found 4864 results. 3122, 244, and 1498 results were found from the EMBASE, Cochrane, and PubMed databases, respectively. The remaining records were not found in the NIH clinical registry Clinical Trials.Gov. We removed duplicate records and unrelated research through keywords such as letter, conference paper and conference abstract. Then we judged titles and abstracts and selected 31 articles. The assessment was performed through reading the entire text. After screening, 23 articles were excluded due to improper experimental planning, or improper research topics, or missing relevant data. Finally, we included eight studies in the review, all of which were randomized controlled trials. However, because of the lack of common outcome metrics, only six studies were finally included in the meta-analysis. Two were clinical studies and four were animal experiments. **Figure 1** displays the selection process.

**Tables 1**, **2** show the general characteristics about the eight studies. Three of them were clinical studies involving 290 subjects (Karim et al., 2022; Koning et al., 2010; Panahi et al., 2017). Five were animal studies involving 211 animals (Sá et al., 2024; Carvalho et al., 2020; Daniel et al., 2021; Mao et al., 2022; Yolanda et al., 2022).

**Table 1:** Characteristics of the human studies included.

Author (Year)	Country/Region	Sample Size (I/C)	Gender (M/F)	Age	Periods of COPD	Course of COPD	Probiotic Species	Dosage	Type of Trial	Intervention Duration	Outcomes
Karim et al. (2022)	United Arab Emirates	100(47/53)	100/0	C: 68.7 ± 4.2 I: 67.1 ± 3.4	stable	No mention	Streptococcus thermo-philus DSM 24731, Bifidobacterial (B. longum DSM 24736, B. breve DSM24732, DSM 24737), Lactobacilli (DSM 24735, DSM 24730, DSM 24733, L. delbrueckii subsp. bulgaricus DSM 24734)	1 capsule/day (Each capsule contains 112 billion live bacteria)	RCT double-blind	16weeks	%FEV1, CRP
Koning et al. (2010)	The Netherlands	30(17/13)	C: 7/6 I: 12/5	C: 63.4 ± 7.4 I: 59.9 ± 13.3	acute exacerbation	No mention	(Ecologicw AAD)	10^9^ CFU/day	RCT double-blind	2weeks	Composition of the major fecal microbiota, Bacterial subgroups, daily defecation score
Panahi et al. (2017)	Iran	60(40/20)	60/0	C: 41.10 ± 4.67 I: 42.22 ± 7.19	stable	C: 29.30 ± 3.75 I: 28.80 ± 5.21	Lactobacillus acidophilus, Lactobacillus bulgaricus, Lactobacillus rhamnosus, Lactobacillus casei, Bifidobacterium breve, Bifidobacterium longum, Streptococcus thermophilusthe.	2 × 10^9^CFU/day; 1 capsule/12h	RCT double-blind	6weeks	%FEV1, CRP

I, intervention; C, control; M, male; F, female; COPD, chronic obstructive pulmonary disease; RCT, randomized controlled trial; %FEV1, percentage predicted forced expiratory volume in 1 s; CRP, C-reactive protein; Ecologicw AAD: a probiotic consisting of multiple species; CFU, colony-forming units.

**Table 2:** Characteristics of the animal studies included.

Author (Year)	Country	Intervention	Sample size	Species	Strain	Gender	COPD model	Probiotic dosage	Probiotic Species	Duration	Outcomes
Sá et al. (2024)	Brazil	Control COPD COPD + Lr GPLG-094 + Lr + CS	7 7 7 7	mice	C57B1/6	male	CS	1 × 10^9^ CFU/0.3mL PBS/mouse	Lr	8weeks	TNF-α, IL-6, IL-1β
Yolanda et al. (2022)	Indonesia	food only Smoke Smoke + Zinc Smoke + probiotics Smoke + zinc + probiotics	6 6 6 6 6	rats	Wistar	male	CS	2.5 × 10^9^ CFU/g	no mention	1week	MDA, iNOS
Carvalho et al. (2020)	Brazil	control COPD + Lr COPD	7 7 7	mice	C57B1/6	male	CS	1 × 10^9^ CFU/0.2mL PBS/mouse	Lr	9weeks	deposition of pulmonary collagen fibers, TNF-α, IL-6, IL-10, IL-1β
Mao et al. (2022)	China	Control COPD APL probiotics BJF	12 12 12 12 12	rats	Sprague Dawley	no mention	CS+LPS	0.9 × 10^10^CFU/kg/day	no mention	5weeks	TNF-α, IL-6, IL-10, IL-1β
Daniel et al. (2021)	USA	LF Control LF DEP HF Control HF DEP HF Control– Probiotics HF DEP– Probiotics	12 12 12 12 12 12	mice	C57Bl/6	male	DEP	7.5 × 10^8^ CFU/day	Bifidobacterium bifidum W23, Bifidobacterium lactis W51, Bifidobacterium lactis W52, Lactobacillus acidophilus W37, Lactobacillus brevis W63, Lactobacillus casei W56, Lactobacillus salivarius W24, Lactococcus lactis W19 and, Lactococcus lactis W58	30days	TNF-α, deposition of pulmonary collagen fibers

GPLG-094, butyrate GPR43 receptor inhibitor; CS, cigarette smoke; MDA, malondialdehyde; iNOS, Inducible Nitric Oxide Synthase; Lr, Lactobacillus rhamnosus; APL, aminophylline; BJF, Bufei Jianpi formula (a traditional Chinese medicine); LF, regular chow; DEP, diesel exhaust particles; HF, high-fat diet.

In two studies, additional interventions were provided to both the intervention and control groups in addition to probiotics/placebo: Yunes Panahi used respiratory medication (salbutamol and fluticasone) plus pulmonary rehabilitation (30 min, 2 times per week); Sarah Daniel used a high-fat diet. In all three human studies, COPD included stable phase (n = 2) acute phase (n = 1). Regarding animal species, three used mice and two used rats.

Of the included literature, two studies had data on %FEV1; two on CRP; four on TNF-α; three on IL-6; two on IL-10; three on IL-1β; and two on pulmonary collagen fiber deposition. One article in the human study and one in the animal study didn’t have common outcome indicators with the other studies.

### 3.2 Study quality

#### 3.2.1 Humans

**Figure 2:**
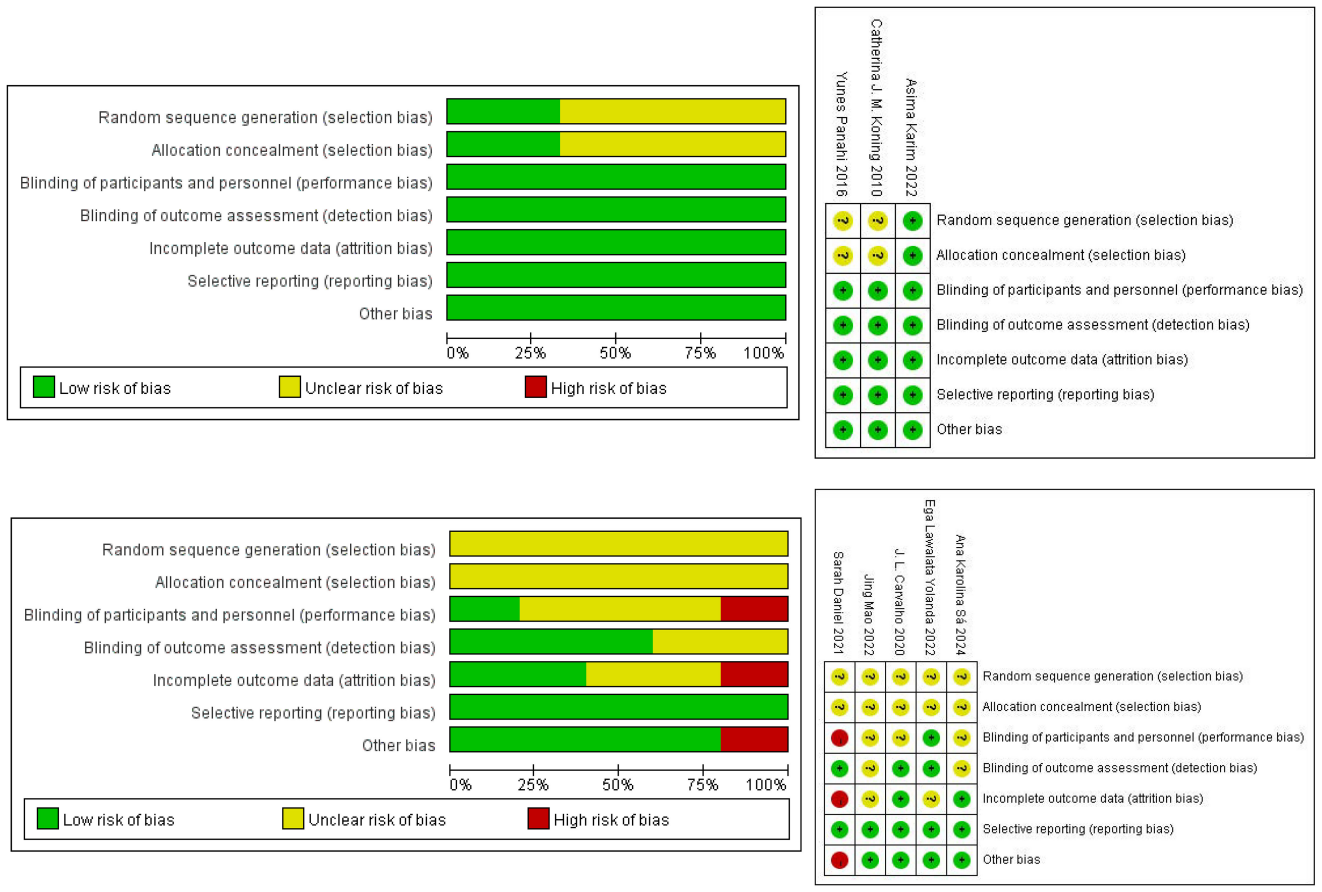
Risk of bias assessment for human and animal studies.

Three studies were randomized. One described the randomization method and allocation concealment in detail, so we determined the selection bias to be “low” risk. Two didn’t have a detailed description, so their risk of selection bias was defined as “unclear”. The risk of performance bias and detection bias was “low”, because all studies were double-blind. None of the included studies had incomplete outcome information, so we considered the risk of attrition bias to be “low”. There was no selective reporting of findings in any of the studies, so we also considered the risk of reporting bias to be “low”, and there was no risk of bias from other sources. The risk of bias for the human studies is presented in **Figure 2**.

#### 3.2.2 Animals

The five animal studies that were eventually included were all randomized. But the exact method of randomization was not specified in the articles, and none of the studies mentioned allocation concealment, so the risk of bias was considered to be “unclear”. For performance bias, Sarah Daniel was not blinded, Ega Lawalata Yolanda was blinded, and whether the others were blinded was not specified. In terms of detection bias, the randomization of the outcome assessment was unclear in two cases, along with whether the evaluators were blinded. In addition, the number of animals in Sarah Daniel’s later experiments didn’t match the number of animals at the start of the modelling, and the reason for this was not explained, so there was a “high” risk of attrition bias. The reporting bias was shown to be “low” risk in all studies. For other sources of bias, only one article was at “high” risk because Sarah Daniel intervened with one cage of animals and analyzed with one animal. The risk of bias for the human studies is presented in **Figure 2**.

### 3.3 Main efficacy of meta-analysis

#### 3.3.1% FEV1

**Figure 3:**
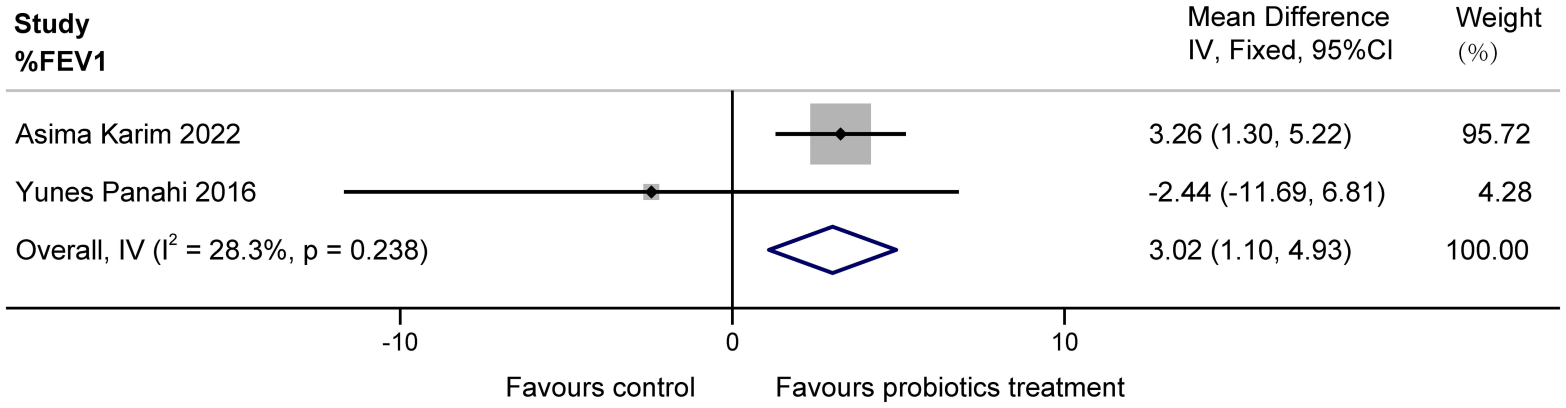
Effect of probiotics on %FEV1 in COPD patients.

The two papers included in this study (Karim et al., 2022; Panahi et al., 2017) examined the effect of probiotics on %FEV1 in patients with COPD. Possibly due to a clerical error, the unit L of FEV1 mentioned by Yunes Panahi in the article was extrapolated to originally be %, as it is unlikely that a person’s exertional expiratory volume in one second is tens of liters. The two studies had a total of 160 subjects, with high confidence interval overlap and no heterogeneity suggested by statistical tests. These data demonstrate that a multi-probiotic supplement significantly improves %FEV1 in stable COPD (MD = 3.02, 95%CI: 1.10 to 4.93, z = 3.085, P = 0.002 < 0.05). The results are presented in **Figure 3**.

#### 3.3.2 Inflammation indicators

##### 3.3.2.1 CRP in patients(mg/L)

**Figure 4:**
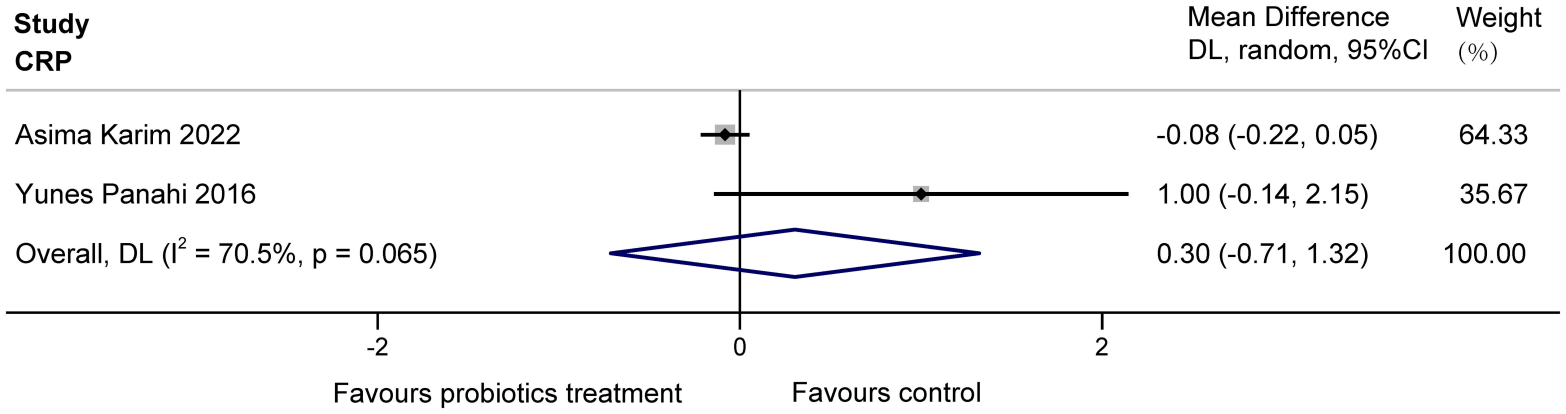
Effect of probiotics on CRP in COPD patients.

The two studies (Karim et al., 2022; Panahi et al., 2017) examined the effects of probiotics on C-reactive protein (CRP) in COPD patients, totaling 160 subjects. Heterogeneity existed between the studies, and it was considered that the heterogeneity may have originated from various aspects, including the choice of sample size, conversion of data, etc. The results found no significant effect of multiple probiotics on stable COPD (MD = 0.30, 95%CI: -0.71 to 1.32, z = 0.587, p = 0.557 > 0.05). The results are displayed in **Figure 4**.

##### 3.3.2.2 TNF-α, IL-1β, IL-6, IL-10 in animals

Jing Mao et al. measured TNF-α, IL-6, and IL-10 in lung homogenates by enzyme-linked immunosorbent assay (ELISA), and the expression of IL-1β in colon tissues by protein blotting. J.L. Carvalho et al. and Ana Karolina Sá et al. measured the levels of TNF-α, IL-1β, IL-6 by bronchoalveolar lavage fluid (BALF). Because of the different ways of measurement and units of the indicators, the SMD method of effect measurement was used.

**Figure 5:**
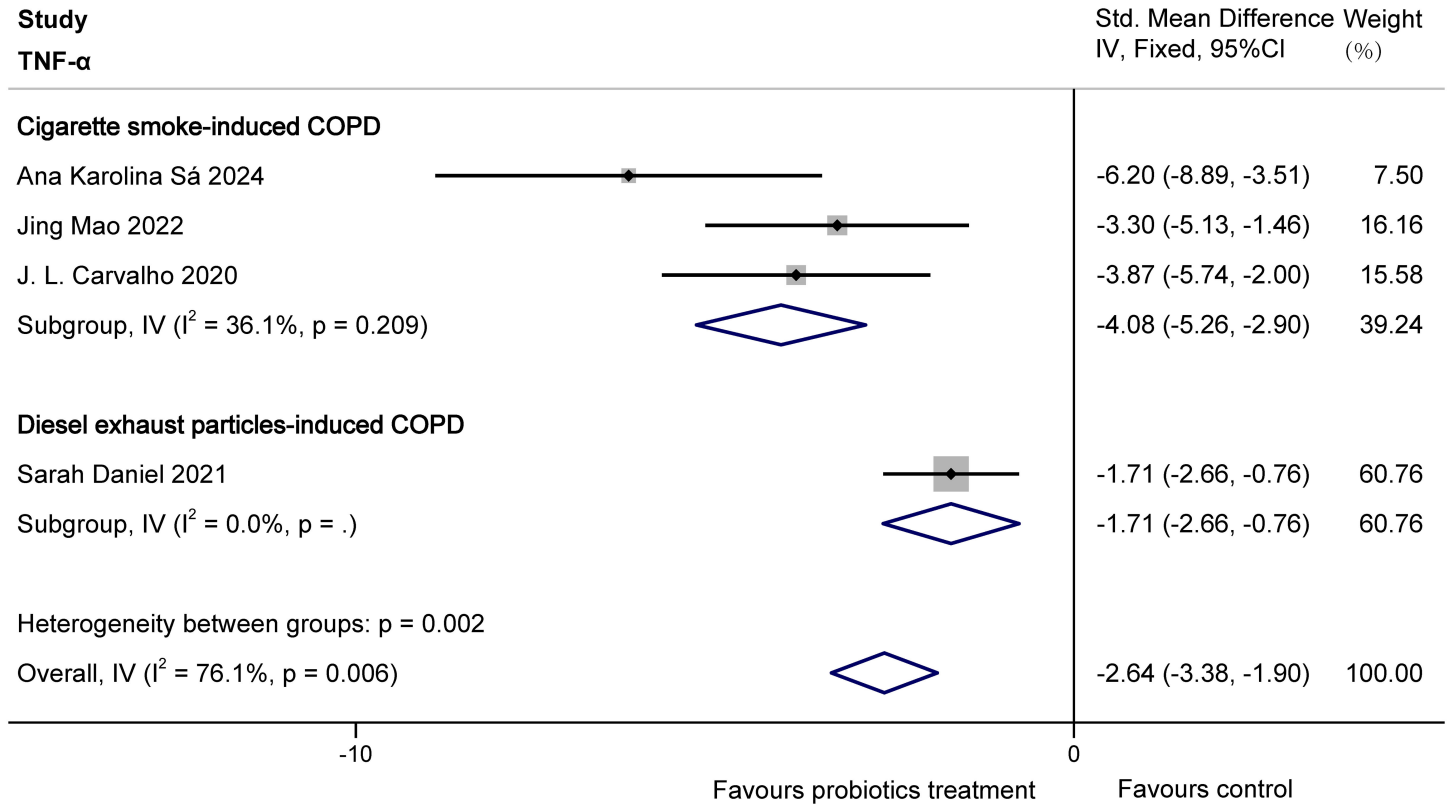
Effect of probiotics on TNF-α in COPD animals.

The four studies involved (Sá et al., 2024; Mao et al., 2022; Carvalho et al., 2020; Daniel et al., 2021) reported the effect of probiotics on TNF-α in COPD animals. There were 64 animals involved. The overall heterogeneity of the study was high (I^2^ = 76.1%>50% and P = 0.006< 0.1 for Q-test). We considered that it might be the difference in modelling methods of COPD animals (three were made by cigarette smoke (CS) modelling, whereas Sarah Daniel used the DEP method), the type and dose of probiotics that led to the difference in results. It is also possible that this is because Sarah Daniel’s study had COPD animals on a high-fat diet regardless of the control or test group, which caused a bias in the data. So, the study was divided into two groups for analysis, based on the specific method of animal modelling employed. Subgroup analyses demonstrated that probiotics significantly reduced TNF-α levels in CS-induced COPD animals (SMD = -4.08, 95% CI: -5.26 to -2.90, z = -6.789, p = 0.000 < 0.05), as well as in DEP-induced COPD animals (SMD = -1.71, 95% CI: -2.66 to -0.76, z = -3.542, p = 0.000 < 0.05). The results are shown in **Figure 5**.

**Figure 6:**
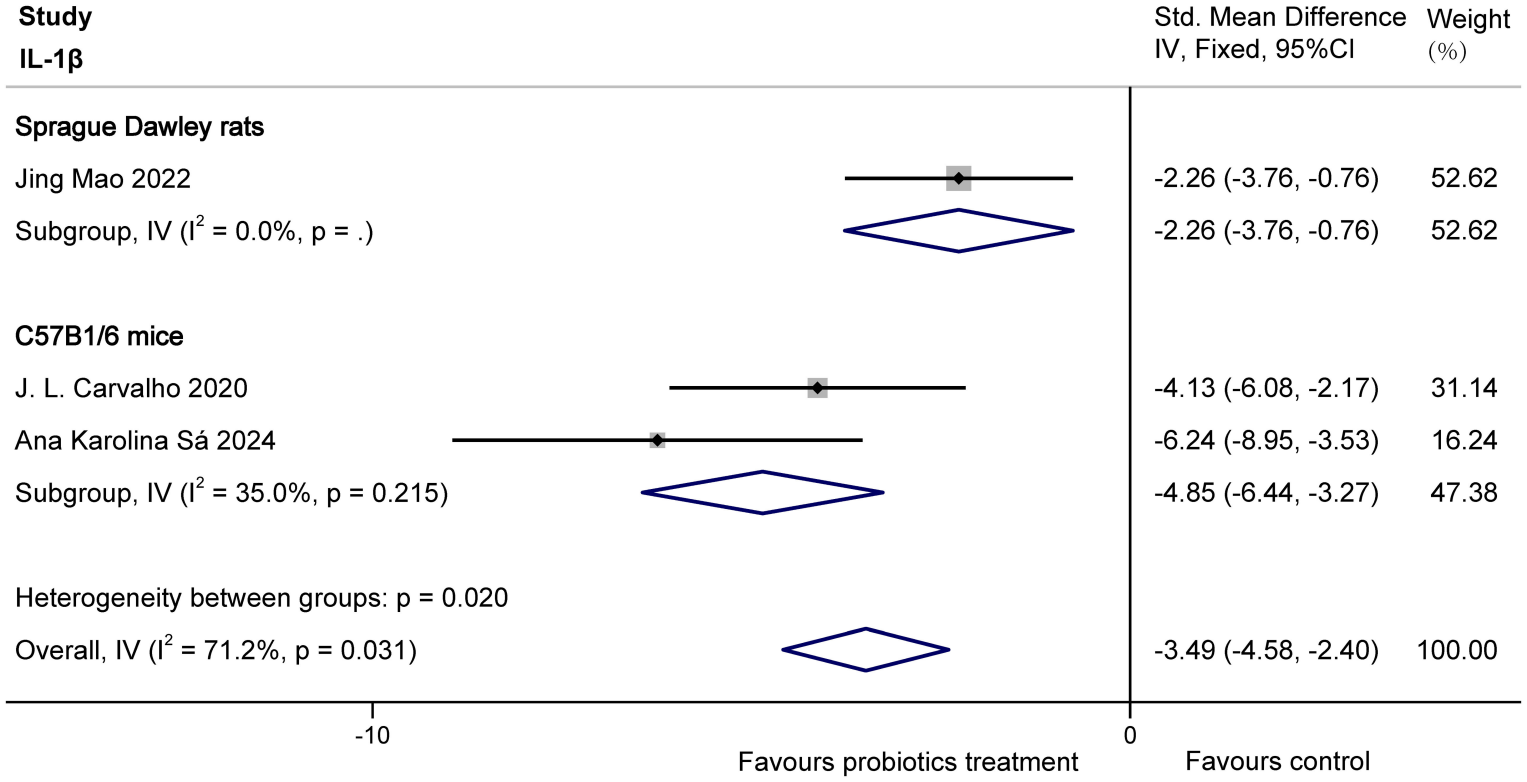
Effect of probiotics on IL-1β in COPD animals.

The three studies (Sá et al., 2024; Mao et al., 2022; Carvalho et al., 2020) examined the effects of probiotics on IL-1β, IL-6 in COPD animals, involving 40 animals. For IL-1β, there was heterogeneity (I^2^ = 71.2% > 50% and P = 0.031 < 0.1 for Q-test), which may have resulted from the different sources of animals or probiotic species (The probiotics used by Ana Karolina Sá and J.L. Carvalho were Lactobacillus rhamnosus, but Jing Mao did not mention the specific type of probiotics). We divided the study into two groups based on animal species to eliminate heterogeneity. Subgroup analyses showed both Sprague Dawley rats (SMD = -2.26 95% CI: -3.76 to -0.76, z = -2.946, p = 0.003 < 0.05) and C57B1/6 mice (SMD = -4.85, 95% CI: -6.44 to -3.27, z = -5.998, p = 0.000 < 0.05) displayed probiotics significantly decreased IL-1β in COPD animals. The results are shown in **Figure 6**.

**Figure 7:**
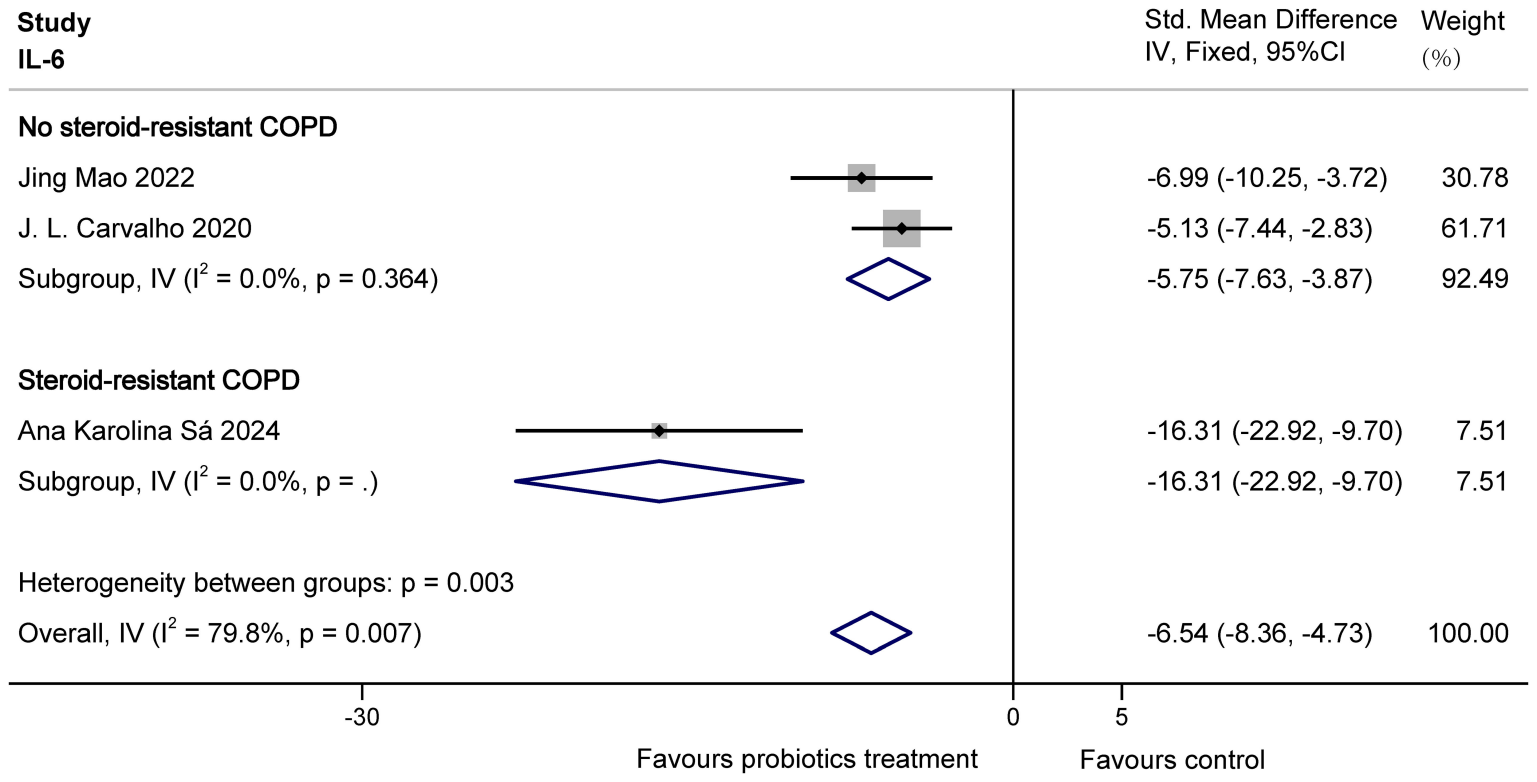
Effect of probiotics on IL-6 in COPD animals.

For IL-6, there was overall heterogeneity. This is probably because the COPD animals modelled by Ana Karolina Sá were steroid resistant with higher levels of inflammatory factors. So based on the presence or absence of steroid resistance, the study was divided into two groups to eliminate heterogeneity. The results of subgroup analysis showed that probiotics were able to significantly decrease IL-6 in both no steroid-resistant COPD animals (SMD = -5.75, 95% CI: -7.63 to -3.87, z = -5.985, p = 0.000 < 0.05) and steroid-resistant COPD animals (SMD = -16.31, 95% CI: -22.92 to -9.70, z = -4.837, p = 0.000 < 0.05). The results are shown in **Figure 7**.

**Figure 8:**
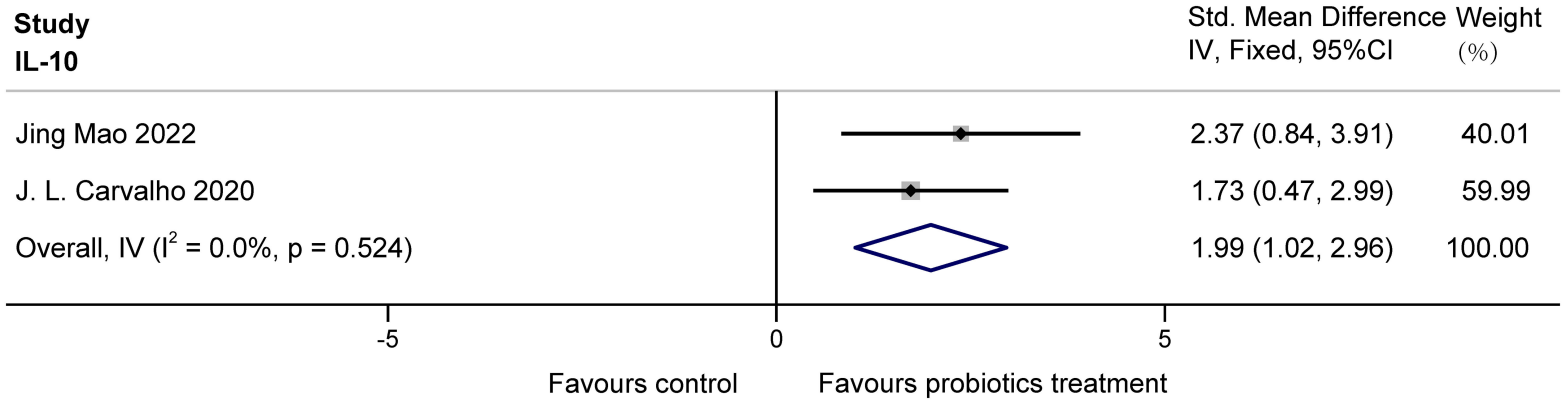
Effect of probiotics on IL-10 in COPD animals.

The two studies (Mao et al., 2022; Carvalho et al., 2020) examined the effects of probiotics on IL-10 in COPD animals, involving 26 animals. For IL-10, there was no heterogeneity. IL-10 was analyzed using fixed effects, which showed that probiotics significantly increased IL-10 in COPD animals (SMD = 1.99, 95% CI: 1.02 to 2.96, z = 4.007, p = 0.000 < 0.05). The results are presented in **Figure 8**.

#### 3.3.3 Collagen fiber deposition in animals

**Figure 9:**
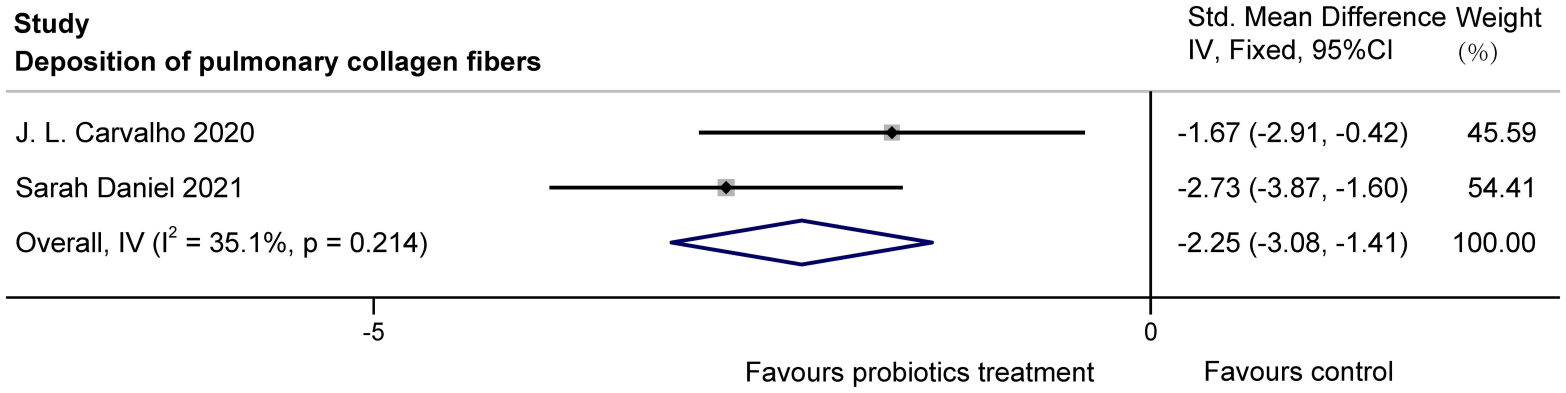
Effect of probiotics on collagen fiber deposition in the lungs of COPD animals.

The two studies (Carvalho et al., 2020; Daniel et al., 2021) examined the effects of probiotics on collagen fiber deposition in the lung bronchioles of COPD animals, with a total of 38 animals involved. J.L. Carvalho et al. used stained lung sections for observation, and Sarah Daniel et al. obtained data by histological scoring of Masson trichrome staining. After statistical analysis, there was no heterogeneity, but because of the difference in the scale, SMD was used. It was found that probiotics significantly reduced collagen fiber deposition in lung bronchioles of COPD animals (SMD = -2.25, 95%CI: -3.08 to -1.41, z = -5.250, p=0.000 < 0.05). The results are presented in **Figure 9**.

#### 3.3.4 Occurrence of adverse reactions in patients

Three studies (Karim et al., 2022; Koning et al., 2010; Panahi et al., 2017) reported patients’ information about adverse reactions occurring while taking probiotics. Among them, Asima Karim et al. mentioned that seven people experienced flatulence and bloating in the probiotic trial group, while the others didn’t.

## 4 Discussion

According to the results, we found that probiotics had a positive effect on COPD in three areas: in terms of lung capacity, probiotics increased %FEV1 levels in COPD patients; In terms of inflammation, probiotics improved inflammation by substantially modulating inflammatory cytokine levels. In animals, this was demonstrated by an increase in IL-10 and a decrease in TNF-α, IL-1β, and IL-6. In addition, in terms of lung structure, probiotics significantly reduced the degree of pulmonary collagen fiber deposition in animals with COPD. Most studies did not report any adverse effects, except for seven individuals in the probiotic group who developed flatulence and bloating in one study. Therefore, we infer that probiotics are an effective treatment for COPD.

Although there is insufficient evidence regarding a therapeutic effect of probiotic supplementation in COPD, the link between the gut microbiota and the lungs is extensively studied. COPD can partially improve two-way regulation between the lungs and the gut in the lung-gut axis. This improvement consists of three main avenues: inflammation, anti-oxidative stress, and improvement of gut microbial composition and metabolites (Wang et al., 2023). The mechanisms by which probiotics ameliorate COPD are mainly explained by these three communication pathways.

Among the inflammatory pathways, there is evidence that lung inflammation leads to intestinal inflammation via circulating inflammatory cells and mediators in the progression of COPD, which in turn exacerbates lung inflammation (Keely et al., 2012; Yanbaeva et al., 2009). In cellular inflammation and immune responses, the NF-κB and MAPK pathways play a crucial role. Probiotics have been shown to reduce pro-inflammatory cytokine levels, upregulate anti-inflammatory cytokines, and alleviate inflammation under various inflammatory conditions. This effect has been associated with reversal of p-IκB protein activation in the NF-κB pathway with inhibition of phosphorylation of proteins p-p38, p-ERK1/2 and p-JNK1/2/3 in the MAPK signaling pathway (Hao et al., 2023; Vincenzi et al., 2023; Faghfouri et al., 2023). At the same time, by preventing bacterial translocation and enhancing the intestinal mucosal barrier (Zeng et al., 2017; Qin et al., 2022), probiotics can break the vicious cycle of pulmonary and intestinal inflammation that promote each other. Therefore, we hypothesize that one mechanism by which probiotics improve COPD is to weaken inflammation.

Probiotics can also ameliorate COPD through the oxidative stress (OS) pathway. Endogenous reactive oxygen species (ROS) are produced by airway epithelial cells and lung immune cells in response to cigarette smoke. ROS enter the gastrointestinal tract through the body’s bloodstream, exacerbating intestinal damage and subsequent lung injury (Wang et al., 2023). ROS-induced OS is known to be an NF-κB-activating factor and stimulates inflammatory responses. Several studies have identified probiotics as a potentially valuable antioxidant (Shi et al., 2019). The use of probiotics reduces the production of ROS and malondialdehyde (MDA) damage and promotes the production of the antioxidants superoxide dismutase (SOD), catalase (CAT) and glutathione (GSH) (Li et al., 2022). As a crucial marker of membrane lipid peroxidation, MDA can disrupt various normal physiological and biochemical pathways. Under normal circumstances, antioxidant enzymes including SOD, CAT and GSH can remove hazardous substances produced during metabolism.

In terms of gut microbial composition and metabolites, COPD patients suffer from gut microbial dysbiosis, which reduces types of symbiotic bacteria that contribute to good health and the synthesis of beneficial metabolites (Li et al., 2021). For example, short-chain fatty acids (SCFAs), produced by fermented fibers from the gut microbiome, can exert anti-inflammatory effects along the lung-gut axis and may reduce chemotaxis and adhesion of immune cells, while increasing the release of anti-inflammatory cytokines and inducing apoptosis (Qu et al., 2022). Butyrate is one of the SCFAs, and strengthens the gut barrier via promoting the close junctions of human colonic Caco-2 cells (Peng et al., 2009). SCFAs are low in the feces of patients with COPD, which may be one of the mechanisms of inflammatory progression. Probiotic supplementation may improve COPD by optimizing the structure of the intestinal flora and increasing beneficial metabolites.

A meta-analysis on whether probiotics can improve COPD has not been studied up to now, but many relevant studies have demonstrated the positive therapeutic effects of probiotics on respiratory disease. Probiotics can reduce lung inflammation. A recent meta-analysis showed that probiotics were effective in reducing serum CRP and improving overall symptoms and inflammatory response in COVID-19 patients (Tian et al., 2023). Our analysis showed that probiotics applied to COPD modelling animals increased the anti-inflammatory cytokine IL-10 and decreased the levels of pro-inflammatory cytokines such as TNF-α, IL-1β and IL-6. Probiotics, on the other hand, had no significant effect on CRP in COPD patients. A prospective cohort study found that CRP levels in patients with clinically stable COPD were stable over 3 months, which suggests that CRP levels tend to steady in stable COPD patients (Park et al., 2023). Due to the lack of relevant data, the clinical trials included in the dataset were all in stable COPD, so we considered the CRP results analyzed reasonable. One of the included trials found that probiotics may mitigate inflammatory progression in COPD by inhibiting the expression of inducible nitric oxide synthase (iNOS) (Yolanda et al., 2022). iNOS is a macrophage-type enzyme that can lead to inflammation by stimulating OS and pro-inflammatory signaling to produce excess NO (Wang et al., 2020). In addition to the studies we included, in a randomized controlled trial, Lactobacillus rhamnosus was shown to be effective in controlling lung inflammation and airway remodeling in mice with asthma-COPD overlap syndrome (Vasconcelos et al., 2023), which is consistent with our results.

Based on the suppression of lung inflammation, probiotics can improve changes in lung structure. Lung inflammation exacerbates fibrous deposition in the bronchi and alveoli. It has been found that DEP induced lung inflammation in mice, which displayed increased macrophages in BALF, an increase in the expression of IL-6, TNF-α, and NF-κB in pneumocytes, and a corresponding increase in the collagen fiber content of alveolar septa (Santana et al., 2019). Our analysis showed that probiotics significantly reduced the degree of pulmonary collagen fiber deposition in COPD animals with respect to lung structure. Improvement in lung structure contributes to improvement in function. Our analyses in lung function have shown that probiotics can improve %FEV1 by approximately 3.02% in COPD patients. A double-blind, parallel trial in patients with allergic asthma found that synbiotics, including Bifidobacterium, significantly increased peak expiratory flow both in the morning and in the evening, suggesting that probiotics may promote respiratory function to some extent (Van de Pol et al., 2011), which supports our results.

Furthermore, in terms of bowel habits, one of the included trials found that the administration of probiotics to antibiotic-treated patients with acute worsening of COPD did not affect either the formation of the predominant fecal microbiota or the appearance of diarrhea-like bowel movements (Koning et al., 2010). Although it did not affect the predominant fecal microbiota, the results of a meta-analysis showed that it was possible to modulate the structure of the intestinal microbiota by promoting the enrichment of Bifidobacterium and Lactobacillus, significantly improving the intestinal barrier function (Zheng et al., 2023). In terms of adverse effects, most of the studies did not find any except for one study in which seven people in the probiotic group developed flatulence and bloating. We believe that this flatulence and bloating may be due to chance.

In terms of heterogeneity, the outcome indicators exhibited variable heterogeneity. Among them, TNF-α, IL-1β and IL-6 of COPD animals showed high heterogeneity, and the possible factors are as follows: (1) Animal species varied across studies. This would lead to adaptations in different experiments, and therefore the animals would have different sensitivities to probiotics. In addition, different COPD modelling methods may lead to differences in disease progression. (2) The measurement methods (ELISA, immunofluorescence analysis) differed between TNF-α, IL-1β and IL-6. The measured values and units also varied widely. The heterogeneity of CRP outcomes in patients with COPD was also high, possibly due to differences in the size of the sample size, conversion of data, and other aspects. The SMD values for the results of our analyses may be slightly larger, presumably related to differences in the values of the raw data, but the heterogeneity between studies is overall low, so it can be ignored.

Although we included high-quality trials in our meta-analysis, there are still some limitations: (1) the number of included trials was limited, including three clinical trials and five animal experiments; (2) there were other confounding factors because the design of each study was different, such as the type and duration of probiotic medication; (3) The experimental outcomes were partially limited, and the long-range efficacy and safety of probiotics applied to COPD patients are still unclear; (4) The human study participants were predominantly male and from a specific geographic region, so the efficacy of our findings for a wider range of Asian and European populations cannot be determined; (5) The main findings were significantly influenced by a single study, such as Asima Karim’s study weight accounting for 95% of the analysis of %FEV1, indicating insufficient stability of the results.

## 5 Conclusions

Probiotics were shown to be important in the treatment of COPD by improving lung function, lung structure and inflammation in our meta-analysis. Therefore, probiotics may be a beneficial addition to COPD. However, in light of the limitations and the potential instability of our results, further high-quality research and large-sample clinical studies are needed to further determine the safety and efficacy of probiotics in the COPD population.

## Data Availability

The datasets presented in this article are not readily available due to privacy or ethical concerns. Requests to access the datasets should be directed to JZ, zhangjp@zcmu.edu.cn.
