# Supplementary material for: Effects of probiotic treatment on patients and animals with chronic obstructive pulmonary disease: a systematic review and meta-analysis of randomized control trials
Source: Front Cell Infect Microbiol. 2024 Sep 11;14:1411222. doi: 10.3389/fcimb.2024.1411222 (PMC11422383; doi:10.3389/fcimb.2024.1411222)
Supplement: Supplementary file 1 [file DataSheet1.pdf]

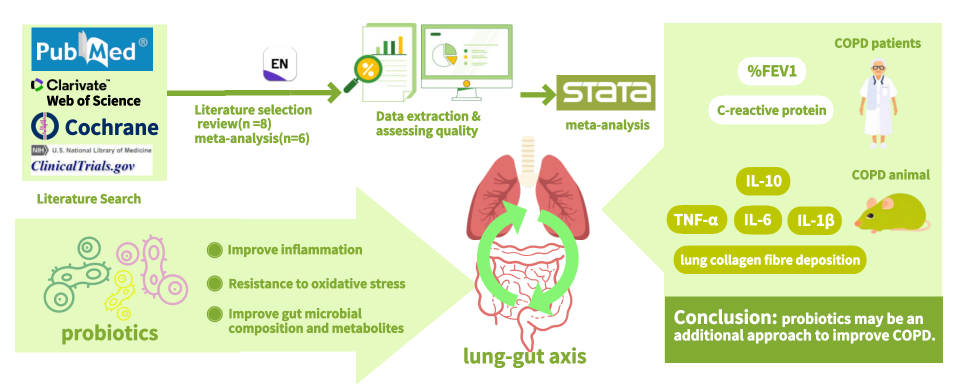

PubMed
Clarivate Web of Science
Cochrane
U.S. National Library of Medicine
ClinicalTrials.gov
Literature Search
EN
Literature selection
review(n =8)
meta-analysis(n=6)
Data extraction &
assessing quality
STATA
meta-analysis
COPD patients
%FEV1
C-reactive protein
COPD animal
IL-10
TNF-α
IL-6
IL-1β
lung collagen fibre deposition
probiotics
Improve inflammation
Resistance to oxidative stress
Improve gut microbial composition and metabolites
lung-gut axis
Conclusion: probiotics may be an additional approach to improve COPD.
